# Medium-Long-Term Radiographic and Clinical Outcomes after Surgical Treatment of Intra-Articular Tibial Pilon Fractures by Three Different Techniques

**DOI:** 10.1155/2018/6054021

**Published:** 2018-03-01

**Authors:** Carlo Biz, Andrea Angelini, Marco Zamperetti, Filippo Marzotto, Silvano Pierluigi Sperotto, Diego Carniel, Claudio Iacobellis, Pietro Ruggieri

**Affiliations:** ^1^Orthopaedic and Traumatology Clinic, Department of Surgery, Oncology and Gastroenterology DiSCOG, University of Padua, Via Giustiniani 2, 35128 Padova, Italy; ^2^Department of Molecular Medicine (DMM), University of Padua, Via Gabelli 63, 35121 Padova, Italy

## Abstract

**Introduction:**

The goal of this retrospective, observational, case series study was to evaluate the medium-long-term clinical and radiographic results of the three most common surgical osteosynthesis techniques used for the treatment of articular tibial pilon fractures: ORIF, MIPO, and EF.

**Materials and Methods:**

A consecutive series of patients with articular pilon fractures who underwent surgery at our institution were enrolled in this study. Fractures were classified according to the Müller AO classification system. Overall outcomes took the following into account: radiographic quality of reduction, evaluated using Ovadia and Beals' criteria; clinical assessment, evaluated using the AOFAS questionnaire; and general health, evaluated with the SF36-v2 Health Survey.

**Results:**

A total of 94 articular pilon fractures (34 type 43-B and 60 43-C) were evaluated with a mean follow-up of 56.34 months (range 33–101). The techniques used were ORIF, MIPO, and EF in 63 (67%), 17 (18.9%), and 14 cases (14.1%), respectively. According to Ovadia and Beals' criteria, good, fair, and poor results were reported in 61 (64.89%), 26 (27.66%), and 7 (7.45%) cases, respectively. The mean AOFAS score was 82.41 for MIPO, 79.83 for ORIF, and 50.57 for EF, respectively. Thirty-nine patients (41.49%) presented early and/or late complications.

**Conclusion:**

Satisfactory outcomes using the three different techniques were reported. In particular, the radiographic outcomes were inversely proportional to the fracture comminutions and statistically different between internal and external osteosynthesis, but comparable between ORIF and MIPO techniques. On the other hand, the clinical outcomes were closely related to the soft tissue conditions and the anatomical reconstruction of the joint.

## 1. Introduction

Tibial pilon fractures are traumatic injuries of the distal part of the tibia involving its articular surface at the ankle joint [1, 2]. They are generally uncommon, representing approximately 7–10% of all tibial fractures and 1–5% of lower limb fractures [3]. Pilon fractures are typically related to severe displacement of fragments, comminution, extensive soft tissue damage with bone exposure and lack of muscle cover, involvement of other skeletal segments, and visceral trauma [3, 4]. Their outcomes are often unsatisfactory with a high percentage of complications [4]. Hence, tibial pilon fractures still represent a significant challenge for orthopaedic surgeons. Presently, nonoperative management using casts or pin traction is advocated by few orthopaedic surgeons and only for nondisplaced articular fractures or in patients who have surgical contraindications because of medical comorbidities, patients with low demand, and select inoperable cases [4, 5]. The objective of operative treatment is to anatomically reduce the fracture fragments in order to restore the congruity of the joint surface and promote bony union and functional recovery with minimal disruption of soft tissues. To this end, several surgical techniques and staged procedure protocols have been proposed for treatment, including open reduction internal fixation (ORIF), minimally invasive plate osteosynthesis (MIPO), and external fixation (EF), often followed by internal synthesis [6, 7]. However, none of these methods seems to be the ideal option for the management of soft tissue injuries and different fracture patterns, as pain, stiffness, and weakness persist for many months, sometimes becoming permanent. The traditional approach, involving an extensive soft tissue dissection to the distal tibia, originally described by Rüedi and Allgöwer [8], has been associated with significant rates of infection and wound dehiscence. Minimally invasive plating osteosynthesis (MIPO) is an alternative that enables indirect reduction and stable fixation with minimal soft tissue complication. However, it seems limited mainly to the treatment of extra-articular fractures (43A), only a few undisplaced articular fractures (43B1-C1), or cases of soft tissue lesions [9]. EF can be very useful as a temporary option for skeletal and soft tissue traction, but as a definitive treatment, it may result in malunion, pin-track infections, and ankle stiffness [10]. The purpose of this retrospective and observational study was to analyse, in a consecutive series of cases, the medium-long-term clinical and radiographic results of the three most common surgical osteosynthesis techniques used for the treatment of articular tibial pilon fractures: ORIF, MIPO, and EF.

## 2. Materials and Methods

### 2.1. Setting and Patients

At our level-I healthcare trauma center, a 1,572-bed multidisciplinary and multispecialty regional university teaching hospital, from January 2006 to January 2013, a consecutive series of patients with diagnosis of tibial pilon fracture were treated surgically. All subjects participating in this retrospective and experimental study received a thorough explanation of the risks and benefits of inclusion and gave their oral and written informed consent to publish the data. The study was performed in accordance with the ethical standards of the 1964 Declaration of Helsinki as revised in 2000.

### 2.2. Inclusion and Exclusion Criteria

All subjects considered in this study had to be older than 18 years, admitted to our Trauma Unit in the selected period, having undergone operative intervention for intra-articular fracture of the tibial pilon, isolated or with associated fibular fracture, having type 43-B or 43-C according to the Müller AO classification [11], and having provided informed consent to participate in the analysis. Specific patient exclusion criteria were as follows: paediatric patients, history of previous foot surgery or trauma, diagnosis of diabetes mellitus, cancer patients receiving chemotherapy and radiotherapy or with bone metastases, and patients with severe orthopaedic comorbidities (severe coxa-arthrosis, gonarthrosis, rheumatological diseases or psoriatic arthritis, diabetic foot neuropathy, vascular insufficiency).

### 2.3. Surgical Techniques, Postoperative Treatment, and Rehabilitation Program

All operative procedures were carried out by one of our trauma team surgeons, the 3 senior authors (I. C., S. S. P., and B. C.), with the help of two different residents of our institution. In all procedures, plexus anaesthesia was performed consisting in a regional block, which involved both sciatic and femoral nerves (biblock). Sedation was used when necessary. Antibiotic intravenous prophylaxis was administered with Cefazolin (1 g 4 times/day) and continued 24 hours after surgery in cases of closed fractures, while Ampicillin and Sulbactam (3 g 4 times/day) were administered and continued for a week in cases of bone exposure. Postoperative antithrombotic therapy (Natrium Enoxaparin) was given until weight bearing.

Without any calcaneal skeletal traction, three types of surgical techniques were performed, each with the patient supine on a radiolucent operating table: (1) traditional ORIF, limited to open reduction and internal fixation; (2) the more recent MIPO technique; and (3) EF, a two-stage management approach involving temporary external fixation followed by definitive open reduction and internal fixation, or a one-stage approach limited to definitive external fixation.

The following fixation methods were used:

- 3.5 mm Locking Compression Plate System (LCP) with additional lag screws (by DePuy Synthes) for ORIF and MIPO;
- Prefix Fixator (by Orthofix, USA) for temporary stabilization EF (two-stage);
- Procallus Fixator or the Hybrid Fixator (by Orthofix) for definitive EF (one-stage).

According to our institutional protocol, the choice to proceed directly with definitive fixation by ORIF or MIPO was based on the absence of any open injuries, good condition of soft tissue or recovery of soft tissue during the days after trauma, and the fracture pattern. Instead, the two-step procedure was chosen on the basis of critical patient condition, soft tissue injuries, important edema, fracture blisters, and fracture pattern. Finally, the decision to convert the EF from temporary to definitive was taken during outcome patient radiographic evaluation depending on the evolution of bone alignment and callus formation at the fracture site. The anteromedial surgical approach was used in all cases treated by the MIPO technique and in the majority of type 43-B fractures treated by ORIF, while the anterolateral approach was generally preferred for those 43-B fractures that were anterolateral partial articular and for type 43-C fractures. During ORIF and MIPO procedures, associated fibular fractures were first stabilized using a lateral approach with 3.5 mm LCP-DCP plates (by DePuy Synthes) or with temporary Kirschner wires. During these procedures, a thigh tourniquet was always applied only in cases of ORIF when any autogenous or allogeneic bone grafting was employed.

During the postoperative period, all patients were followed up and plain radiographs were obtained in the immediate postoperative time and at 1 and 3 months after surgery and every 6 months until fracture healing occurred, according to our standard aftercare algorithm. Partial weight bearing and exercises were started after callus formation, while full weight bearing was allowed after bone union. Finally, the external fixators were removed after bone union.

### 2.4. Patient Assessment

Data collection was retrospectively performed during a period of 24 months, from September 2014 to August 2016, by an external and independent investigator (M. F.) not involved in the patients' treatment. Consulting the computerized archives of our hospital, we found that the following information was recorded: patients' age and gender, trauma mechanism, fracture type, closed or open (classified according to the Gustilo-Anderson criteria [12]), soft tissue condition after trauma, surgical technique performed, and time elapsed between trauma and surgery. The clinical and radiological analyses were carried out, respectively, by two independent researchers who were not directly involved in the patients' surgical treatment (C. D. and Z. M.).

### 2.5. Radiographic Outcome Measures

Radiographic data were obtained by reading the radiographic computerized images available in the computer system of our institute. The radiographic evaluation comprised the analysis of conventional radiographs including anterior-posterior ankle radiographic computerized images of all patients taken preoperatively, postoperatively, 1, 3, and 6 months after surgery, and at final follow-up. Further, a diagnostic LCD CORONIS 5 MP display monitor (produced by Barco, Rome, Italy) was used to determine the fracture patterns and carry out the radiological analysis. The fractures were classified according to the Müller AO classification system [11], while the quality of operative reduction, the fractures' evolution, and healing were evaluated by analysing postoperative and last follow-up radiographs according to Ovadia and Beals' criteria [13]. These data are reported in Table 1.

### 2.6. Clinical Outcome Measures

At the time of this study, a phone contact was attempted for all patients who met inclusion criteria, and a follow-up appointment was fixed. Patients who returned were examined, and clinical results were measured with validated questionnaires. To quantify pain and functional disability, the American Orthopaedic Foot and Ankle Society (AOFAS) ankle-hindfoot scale questionnaire [14] was used. Overall physical and mental health were evaluated with the SF-36v2 (MOS short form questionnaire version 2) [15]. As the AOFAS ankle-hindfoot scale takes into account several subjective parameters, which vary according to patient age, our cohort was divided into two groups based on the median age of our sample, ≤ and >52 years. Finally, during the analysis, any early and late complications were recorded and then classified as being minor or major complications. Minor complications were defined as those successfully treated without affecting bone healing, while major complications as those requiring more extensive treatment and affecting bone healing.

### 2.7. Statistical Analysis

Statistical analysis was performed by an independent statistician (F. A. Ch.) from the Department of Statistics at our university, blinded to the type of technique treatment. Analysis of data was performed using SPSS statistical software. The relationship between treatment and AO fracture classification, type of fracture (open or closed), and conditions of skin and soft tissue complications after surgery were analyzed with the Chi-Square Test or Fisher Exact Test. The results of the SF-36v2 survey were used to compare treatment groups and the groups divided according to AO classification. The Wilcoxon Test was used for comparison between two groups, and the Kruskal-Wallis Rank Sum Test was used when more than two groups were involved. When the results of the Kruskal-Wallis Test were statistically significant, a post hoc Dunn Test of multiple comparisons was performed. Mean physical and mental components of SF36 were compared to mean data of the Italian population (confidence interval 95%) [16].

## 3. Results

### 3.1. Patient Data

One hundred and five patients with diagnosis of single tibial pilon fractures were treated surgically in our institution between January 2006 and September 2013. Among the 105 fractures investigated, 11 fractures were excluded from the study because the patients did not meet the inclusion criteria: one patient was undergoing chemotherapy for lung cancer, 4 patients had a history of diabetic foot, one was an amputee, 2 patients suffered severe ipsilateral coxarthrosis, and 3 patients presented severe ipsilateral gonarthrosis. Hence, a total of 94 enrolled patients (94 fractures) were included in this retrospective analysis, and all were available for clinical and radiographic follow-up at an average of 56.34 months (range 33–101). The patients' details are summarized in Table 2. They were 29 (30.85%) females and 65 (69.15%) males. The mean patient age at time of surgery was 52.44 years (range 19–90) with a median of 52 years. Trauma mechanisms were car accident (36.16%), accidental fall (28.73%), fall from height at workplace or at home (24.47%), direct trauma like crushing (5.32%), sports injury (4.26%), and firearm trauma (1.06%).

According to the Gustilo-Anderson classification [10], there were 7 (7.45%) Grade I, 6 (6.38%) Grade II, and 2 (13.2%) Grade III open fractures. With regard to soft tissue conditions, 41 (43.62%) patients showed soft tissue damage such as edema, severe swelling, blistering, skin abrasion, and open wounds. The 53 patients with intact skin were treated by a one-stage procedure protocol in 36 (67.92%) cases, a two-stage procedure protocol in 14 (26.41%) cases, and definitive EF in 3 (5.66%) cases. The 41 patients with soft tissue injuries were treated by a one-stage procedure protocol in 5 (12.2%) cases, a two-stage in 25 (60.97%) cases, and definitive EF in 11 (26.83%) cases. The surgical techniques and treatment methods were performed as follows: ORIF in 63 cases (67%), MIPO in 17 (18.9%), and EF in 14 (14.1%). The 79 closed fractures were treated by ORIF in 58 cases (73.42%), MIPO in 16 cases (20.25%), and definitive EF in 5 cases (6.33%), while the 15 open fractures were treated by ORIF in 5 cases (33.33%), MIPO in 1 case (6.67%), and definitive EF in 9 cases (60%).

### 3.2. Radiographic Outcomes

**Figure 1:**
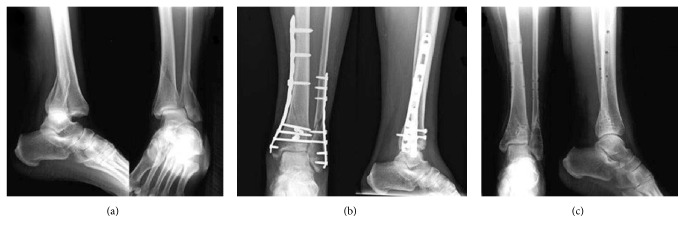
*Case N*°* (42) of Table 2:* a 41-year-old woman with a 43-B1 closed fracture treated with* MIPO technique:* (a) preoperative radiographic image; (b) postoperative radiographic image at 1-month follow-up; (c) radiographic aspect at 39-month follow-up after implant removal.

**Figure 2:**
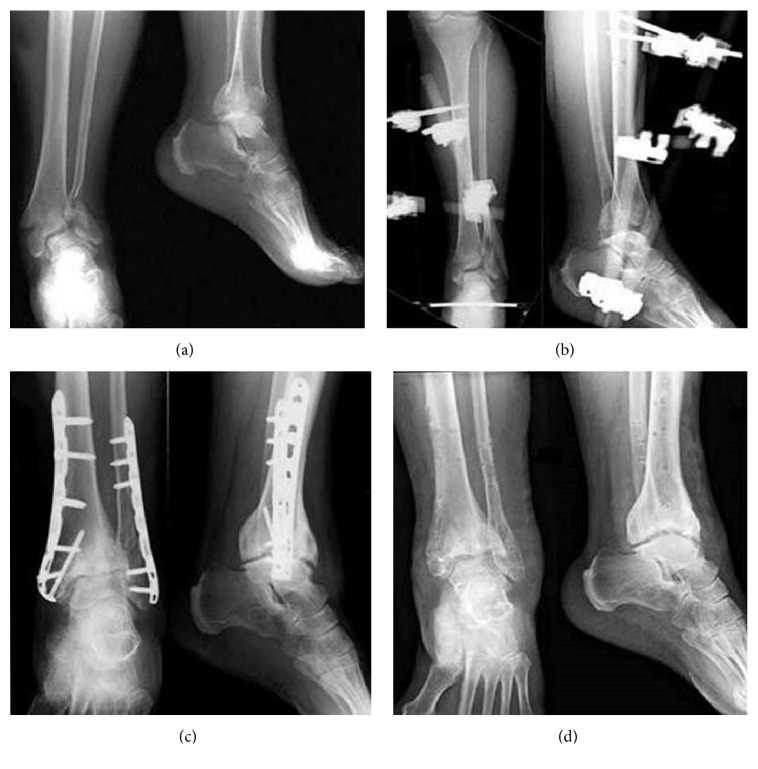
*Case N*°* (47) of Table 2:* a 68-year-old man with a 43-B3 closed fracture treated with two-stage* ORIF technique:* (a) preoperative radiographic image; (b) after prefix implant, radiographic image; (c) definitive implant after 16 days, 1-month follow-up; (d) radiographic aspect at 37-month follow-up after implant removal.

**Figure 3:**
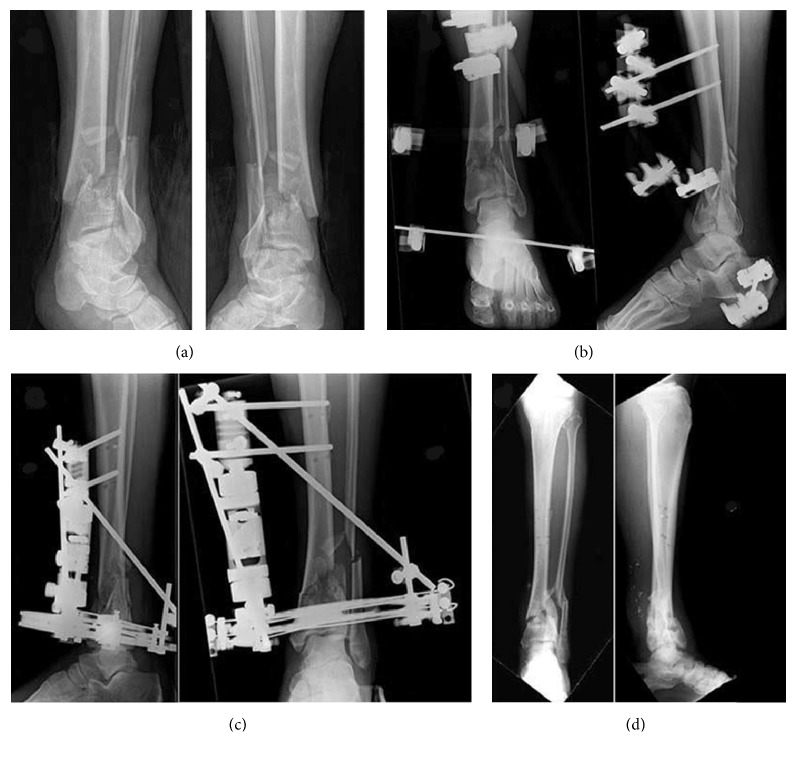
*Case N*°* (34) of Table 2:* a 43-year-old man with a 43-C1 open fracture (Gustilo II) treated with* hybrid external fixator:* (a) preoperative radiographic image; (b) after prefix implant, radiographic image; (c) definitive EF implant after 1 week, radiographic image; (d) radiographic aspect at 57-month follow-up after implant removal.

According to the Müller AO fracture classification [11], there were 34 (36.17%) type 43-B and 60 (63.83%) type 43-C fractures. Closed fractures were reported in the majority of the cases, 79 (84.04%), while open fractures only in 15 (15.96%) cases. According to Ovadia and Beals' criteria [13] at the last follow-up, the radiographic results of our cohort were classified as good, fair, and poor in 61 (64.89%), 26 (27.66%), and 7 (7.45%) cases. For each technique, they were good in 45 cases (71.42%), fair in 16 (25.39%), and poor in 2 cases (3.17%) when treated by ORIF; good in 14 (82.35%) and fair in 3 cases (17.65%) when treated by MIPO; finally, good in 2 cases (14.28%), fair in 7 (50%), and poor in 5 (35.71%) when treated by EF. The radiographic outcomes of our cohort are summarized in Table 3, while one case for each treatment technique is reported in Figures 1, 2, and 3.

### 3.3. Clinical Outcomes

The mean overall AOFAS ankle-hindfoot score was 73.3 (18–100) with a standard deviation of ±20.8. The results of the AOFAS ankle-hindfoot score calculated in the 2 groups according to the median age of the sample (≤ and >52 years) were 81.8 (±15.51) and 70.6 (±24.24), respectively. Patients treated by ORIF, MIPO, and EF as a definitive treatment achieved a mean score of 79.83, 82.41, and 50.77, respectively. The mean (±SD) AOFAS score of patients who did not develop complications was higher than those of patients who developed complications, 81.2 (±18.66) versus 68.31 (±22.86). Regarding the SF-36v2 survey, the mean PCS score of our cohort was 44.3 (range 62–17.7): 45.72 for ORIF, 49.02 for MIPO, and 33.18 for EF. The mean MCS score of the sample was 45.76 (range 60.2–17.7): 46.13 for ORIF, 49.39 for MIPO, and 39.73 for EF. The clinical outcomes are also summarized in Table 3.

Complications were reported in 39 patients (41.49%). There were 20 early complications (21.28%), all minor: 12 (12.77%) superficial infections including pin-track infection and 5 (5.32%) wound dehiscence, which were effectively resolved with local therapy and oral antibiotics; 3 (3.19%) cases of deep venous thrombosis which were treated successfully first by LMWH and then by warfarin therapy. Late complications occurred in 19 patients (20.21%), including 17 major ones (18.08%): 7 (7.44%) delayed union, 4 (4.26%) osteomyelitis, 3 (3.19%) malunions, 3 (3.19%) loss of reduction, and 2 (2.13%) minor complications, such as Sudeck syndrome. No cases of compartment syndrome or secondary bone necrosis were noted, neither rupture of metalwork nor anchorage elements of the EF, while the 3 patients having loss of reduction necessitated revision of their Procallus Fixators. Hence, in our series, 22 cases (23.40%) experienced minor complication, while 17 (18.09%) experienced major complications (Table 4).

## 4. Discussion

The purpose of the present retrospective, nonrandomized, case series study was to evaluate the medium-long-term clinical and radiographic results of the three most commonly performed surgical techniques used to treat tibial pilon fractures [1, 4]: ORIF, MIPO, and EF. In line with other comparable studies [1, 15], the most common technique used in our cohort was ORIF, which was performed more frequently in both type B (73.53%) and C fractures (58.33%) (*p* < 0.05). As the use of the definitive external fixation procedure or the two-stage procedure protocol, in combination with ORIF, represents the gold standard of treatment of pilon fractures in cases of associated soft tissue lesions [17–27], the choice between the one- and two-stage procedure standard protocol in our case series was made in the majority of cases on the basis of the state of the soft tissues (*p* < 0.05) according to our institutional protocol and in a few cases on the basis of the preferences and experience of the surgeons involved in the operations. Further, the choice of using the Procallus Fixator or the Hybrid Fixator for definitive treatment (one-stage procedure) instead of the circular frame was mainly made for biomechanical reasons. In contrast with other authors [28, 29], we believe that the traditional ring fixator is not indicated for the treatment of these fractures, as it has only a mechanical stabilization function without the dynamization exerted by the elasticity of the K-wires during early weight bearing, which is not permitted in these injuries. Further, the Hybrid Fixator is preferred to avoid the rings' application on the proximal leg, which is poorly tolerated by patients.

Despite the severity of the injuries in our sample and their poor prognoses [19], satisfactory radiographic and clinical outcomes were achieved. Regarding radiographic aspects, Ovadia and Beals scores were compared with the AO classification of fractures, revealing a statistically significant difference (*p* < 0.05), as reported by other authors [24, 25]. In accordance with the recent literature [24, 25, 30, 31], joint reconstruction was achieved significantly better by ORIF and MIPO techniques compared to EF (*p* < 0.05), while no statistically significant differences were reported between ORIF and MIPO (*p* > 0.05). In our series, the quality of joint reconstruction depended on the type of fracture. In agreement with the data reported by Korkmaz et al. [31], the reconstruction quality of open fractures was worse. Specifically, good Ovadia and Beals scores were obtained in closed (68.57%) and open (40%) fractures, while poor results were recorded only in a few cases (5.71%) of closed fractures versus 30% for exposed ones (*p* < 0.05).

Regarding clinical outcomes, the average AOFAS score of our cohort was 76.59 points (range 20–100), with 54.25% of our patients scoring between 80 and 100 points, while only 1.06% of our patients achieved very low results (0–20 points). Further, when AOFAS average scores were evaluated in comparison to the AO classification fracture types and their subgroups, no statistically significant difference among them was noted (*p* > 0.05). However, when AOFAS average scores were evaluated in comparison to the 3 different surgical techniques, the scores reported for ORIF (79.83), MIPO (82.41 points), and EF (50.57) were found to be of borderline significance (*p* = 0.0518). Although comparable results are reported in similar studies [31–33], we believe the lack of statistical significance in relation to the surgical technique used in our cohort may be attributable to several subjective parameters that the AOFAS questionnaire takes into account. This score seems to reflect the age of the patients and associated possible comorbidities rather than the true posttrauma foot and ankle clinical situation; the AOFAS average scores were statistically significant only in patients younger than 52 years (*p* < 0.05). As found in other studies [9, 10], AOFAS scores in our cohort were predictably lower in patients who developed postoperative complications compared to patients who did not (*p* < 0.05). Specifically, the correlation assessed between AOFAS and Ovadia and Beals scores was statistically significant (*p* < 0.01). In agreement with Korkmaz et al. [31], our data show that also the functional AOFAS index was closely related to the quality of joint reconstruction.

Dividing the cohort based on the type of treatment, the results obtained by the SF-36v2 questionnaire were higher on average in the group of patients operated by MIPO (49.02 points), slightly lower in that operated by ORIF (45.72), and lower (33.18) in the group treated by EF for the physical component of the score (PCS). The results were similar for the mental component summary (MCS). No significant differences between mean PCS and MCS scores were reported between the three techniques (*p* > 0.05), neither were significant differences found between PCS, MCS, and the AO classification fracture types and subtypes (*p* > 0.05). Similar results are reported in the literature [21, 34–36], obtaining a statistically significant difference only by dividing the sample into age groups and comparing the results with the general population [17]. Finally, the average score for the physical component of the SF-36v2 of the patients in our sample (44.17 points) was compared with the average score of the same index of the Italian population (50 points) [16]. In the literature, some studies have found similar results, getting even lower scores when taking into account only the most serious fractures [18, 20–23].

Because of the high incidence of complications with poor outcomes reported in pilon fracture management [19, 26, 27, 30, 31], several authors [19, 27, 30] have tried to make their surgical approaches less invasive to limit damage of soft tissues. In our cohort, 39 patients (41.49%) showed early and/or late complications, rates comparable to those described in the literature [27, 31]. However, 22 cases (23.40%) experienced early minor complications, including superficial infections, pin-track infections, or wound dehiscence, which were effectively treated by periodic medication, sometimes in association with oral antibiotic therapy for a few days. It is well known that pin-track infection is a frequent complication of this surgical treatment that rarely leads to osteomyelitis, neither does it affect the final outcome [19]. On the contrary, 17 cases (18.08%) experienced major complications, which required further treatment, including nonunion, deep infections, and osteomyelitis. Comparing these findings with the three surgical techniques adopted, they were statistically significant (*p* < 0.05). Specifically, MIPO was correlated to lower complication rates, as this technique is less invasive and preserves soft tissue, which is often seriously damaged before surgery [1, 37, 38]. However, minimally invasive techniques may not permit adequate visualization of the joint surface and achieve an anatomic reduction, with the frequent result of inadequate joint surface reconstruction [19]. Hence, we believe MIPO is not indicated for severely comminuted articular fractures for which ORIF is recommended except in cases of soft tissue lesions when temporary or definitive external fixation should be used.

Selection and assessor biases are always possible, primarily in cases of nonconformity of a standardized protocol. Our patients were treated individually, according to our institutional protocol, sometimes influenced by one of three surgeons' preferences. Several potential limitations and some biases may have influenced this case series study, mainly linked to its retrospective design and the consequent lack of randomization and an identified control group. At the time of the operation no preoperative CT scan planning was performed, neither did our study protocol provide CT scans in the postoperative period for radiographic evaluation. As this report is retrospective, it was difficult in the aftermath to precisely classify the soft tissue conditions at the time of trauma only on the basis of patients' clinical notes. Hence, we recorded only the presence or absence of soft tissue damage rather than using a recognized international classification, which would have been difficult to apply retrospectively. Further, no bone grafting was used for the treatment of the cases included in this study, although its use would probably have improved our results for the most severe injuries. There was also variation in postoperative management in our trauma center, with three different orthopaedic consultants and several residents managing patients at any given time. Further, the lack of fracture type homogeneity in the three treatment groups influenced the PCS and MCS scores values so as to make comparison difficult, without apparent statistical significance. Another possible bias is severity of injury and subsequent choice of fixation: in particular, the use of MIPO for less challenging cases and subsequent good results. The use of EF in the two-step technique for the more complex soft tissue injuries could explain the high infection rate we report, including pin-tract infections, even if they were resolved in all cases by antibiotic therapy. Another potential limitation is that we did not collect the education levels and jobs of our patients, which is reportedly associated with better ankle score when it is high, in particular for white-collar jobs [34]. In addition, the few EF readjustments performed during the patients' outcome activities were not always reported in the clinical notes. However, in comparison to other retrospective recent studies [9, 38–40], our report presents a large sample size (94 patients), taking into account the relatively low incidence of pilon fractures (1% of all lower extremity fractures); it is also one of the largest monocenter cohorts studied, with only three treating surgeons. Another strength of this study is the good quality of data reported from our hospital database, recorded according to our standard aftercare algorithm, which were collected by an independent investigator. The analysis of the clinical and radiographic outcomes was carried out separately by two other researchers and finally analyzed by an independent statistician, blinded to the type of technique treatment in order to reduce bias. Further, the medium-long-term follow-up period allowed the assessment of different functional aspects, as convalescence tends to be long. To perform a comparison of the various possibilities of treatment for the same type of fracture, a prospective trial with a strict standardized protocol, validated functional outcome measurements and a control group would yield stronger evidence.

## 5. Conclusion

Although the documented complications are mostly minor with percentages comparable to those described in the available literature, this case series study reports satisfactory results using the three different techniques for the treatment of articular pilon fractures. In particular, the radiographic outcomes were inversely proportional to the fracture comminutions, statistically different between internal and external osteosynthesis, but comparable between ORIF and MIPO techniques. On the other hand, the clinical outcomes were closely related to the soft tissue conditions and the anatomical reconstruction of the joint. In summary, as can be expected, our findings show that the most severe injuries and the worst radiographic results were related to the worst functional and patient reported outcomes. However, the proper surgical treatment must be carefully chosen for these challenging fractures.

## Figures and Tables

**Table 1:** Ovadia and Beals score. The score sets a value of 0 for poor results, 2 for fair results, and 3 for good results. Adding the six variables, if the score goes from 0 to 6, it is poor; if it goes from 7 to 12, it is fair; if it goes from 13 to 18, it is good.

Classification of fracture reduction			
	Good	Fair	Poor
Malleolus			
Lateral	Anatomical or ≤1.0 mm displacement	2.0–5.0 mm displacement	>5.0 mm displacement
Medial	≤2.0 mm displacement	2.0–5.0 mm displacement	>5.0 mm displacement
Posterior	Proximal displacement ≤2.0 mm	Proximal displacement 2.0–5.0 mm	Proximal displacement >5.0 mm
Mortise widening	≤0.5 mm	0.5–2.0 mm	>2.0 mm
Talus			
Tilt	≤0.5 mm	0.5–1.0 mm	>1.0 mm
Displacement	≤0.5 mm	0.5–2.0 mm	>2.0 mm

**Table 2:** Patient and fracture characteristics of our cohort.

N°	Gender	Age	FW-UP (months)	AO	Technique	Ovadia	AOFAS	SF36 (PSC)	SF36 (MCS)	Gustilo	TISS.DAMAGE
(1)	M	49	69	C2	ORIF	Fair	47	22.8	35.6		Yes
(2)	M	70	54	B3	ORIF	Fair	78	45.2	44.3		
(3)	F	46	47	B1	MIPO	Good	95	55.3	46.8	I	Yes
(4)	F	49	39	C2	MIPO	Good	100	55.5	57.2		
(5)	F	20	65	B1	ORIF	Good	78	47.1	44.1		
(6)	M	28	49	C1	ORIF	Good	90	55.37	50.8		
(7)	F	22	34	C2	ORIF	Good	94	38.5	42.3		Yes
(8)	M	51	45	C3	EF	Fair	53	20.5	55.1		
(9)	M	20	67	C2	ORIF	Good	89	45.6	30.1		Yes
(10)	M	61	46	C3	ORIF	Good	96	55.5	60.2		Yes
(11)	F	20	42	C1	ORIF	Good	100	54.4	60.0		
(12)	M	79	72	C1	ORIF	Fair	41	21.6	31.2		Yes
(13)	F	70	60	C1	ORIF	Good	77	44.3	46.3		
(14)	M	41	58	C3	ORIF	Fair	58	32.9	50.8	I	Yes
(15)	M	20	46	B1	ORIF	Good	96	51.3	45.8		
(16)	M	59	54	B3	ORIF	Good	93	57.0	54.3		
(17)	M	49	39	B3	ORIF	Good	95	50.8	55.37		
(18)	M	69	43	B2	ORIF	Good	98	51.0	44.5		
(19)	M	43	50	C2	MIPO	Good	97	50.1	53.6		
(20)	M	60	47	C2	ORIF	Good	98	55.5	60.0		
(21)	F	64	79	C3	ORIF	Fair	89	46.8	57.1		Yes
(22)	M	27	48	C1	ORIF	Good	100	55.6	60.9		
(23)	F	65	42	C1	ORIF	Good	88	46.2	55.9		
(24)	M	64	66	C3	EF	Poor	20	20.6	17.7	III	Yes
(25)	M	37	38	C2	MIPO	Good	71	35.2	37.1		
(26)	F	52	74	C2	ORIF	Fair	61	43.9	44.1		Yes
(27)	F	33	37	C2	ORIF	Good	100	57.0	55.5		
(28)	M	85	100	B2	MIPO	Fair	52	33.3	35.5		
(29)	F	19	44	B2	MIPO	Good	90	44.7	46.8		
(30)	F	22	67	C2	ORIF	Poor	44	21.7	22.5		Yes
(31)	M	82	63	C3	MIPO	Good	78	56.0	53.4		Yes
(32)	F	52	56	C3	ORIF	Fair	50	25.7	47.2	I	Yes
(33)	M	29	39	B2	ORIF	Good	90	49.8	33.2		Yes
(34)	M	43	57	C1	EF	Poor	60	38.0	28.0	II	Yes
(35)	M	83	97	C3	EF	Poor	29	17.7	27.6	III	Yes
(36)	M	56	44	C1	ORIF	Good	98	59.9	58.0		
(37)	M	78	76	B3	ORIF	Poor	39	30.2	19.2	I	Yes
(38)	F	88	34	C3	ORIF	Fair	45	24.5	46.1		
(39)	M	64	46	C2	MIPO	Good	88	48.9	51.6		
(40)	M	23	76	C3	ORIF	Good	96	57.8	58.9		Yes
(41)	M	63	35	C1	ORIF	Good	82	43.0	51.8		
(42)	F	41	39	B1	MIPO	Good	100	62	60.9		
(43)	M	45	39	C2	ORIF	Good	86	48.1	42.5		Yes
(44)	F	74	37	C1	MIPO	Good	90	58.2	53.0		
(45)	M	42	70	B2	EF	Fair	68	46.7	36.1		Yes
(46)	M	34	61	B2	ORIF	Good	96	53.2	56.2		
(47)	M	68	37	B3	ORIF	Fair	65	37.9	35.4		
(48)	M	81	45	C2	ORIF	Fair	59	23.8	32.2		Yes
(49)	F	60	69	B3	ORIF	Good	78	42.8	42.3		Yes
(50)	F	36	88	B2	MIPO	Good	58	42.1	44.8		
(51)	F	52	41	C2	ORIF	Good	96	57.9	44.5		
(52)	M	87	67	B3	ORIF	Fair	78	43.6	49.2		
(53)	M	24	33	B3	ORIF	Fair	48	35.9	21.1	I	Yes
(54)	M	67	67	C1	ORIF	Good	68	23.3	42.5		Yes
(55)	M	19	37	B3	ORIF	Good	82	55.9	35.1		
(56)	M	72	35	B3	ORIF	Good	89	56.9	56.33		
(57)	M	39	85	C3	ORIF	Fair	70	47.8	58.2		Yes
(58)	M	23	42	C2	MIPO	Good	90	57.3	58.9		
(59)	M	88	70	C2	EF	Poor	38	19.5	25.7	II	Yes
(60)	M	84	83	C2	EF	Fair	70	44.5	49.5	I	Yes
(61)	F	60	67	C1	ORIF	Good	88	44.9	51.7		
(62)	F	46	40	B3	ORIF	Fair	65	45.8	43.0		Yes
(63)	M	83	71	C3	EF	Fair	47	36.4	45.2		Yes
(64)	M	21	51	B3	ORIF	Good	100	57.0	60.1		
(65)	M	47	61	C3	MIPO	Good	94	56.2	59.1		Yes
(66)	M	61	68	C2	ORIF	Good	83	43.6	44.5		
(67)	M	82	52	C1	ORIF	Good	62	43.9	46.9		Yes
(68)	M	52	93	B3	MIPO	Fair	60	39.8	41.2		
(69)	F	29	36	C2	EF	Good	90	57.4	57.2		
(70)	M	32	59	C2	ORIF	Good	92	44.9	42.1		
(71)	M	72	38	C1	MIPO	Fair	75	40.5	39.4		
(72)	F	35	48	C2	MIPO	Good	76	42.1	44.6		
(73)	M	76	35	C1	ORIF	Good	98	37.0	44.0		
(74)	M	75	40	B1	MIPO	Good	87	56.2	55.8		
(75)	F	38	60	B2	ORIF	Good	100	57.6	51.9		
(76)	F	90	101	B3	ORIF	Fair	54	38.5	23.2		Yes
(77)	M	30	45	B3	ORIF	Good	90	60.1	56.5		
(78)	M	40	49	C2	ORIF	Good	98	57.6	39.8		
(79)	M	75	62	C2	ORIF	Good	87	46.9	55.9		
(80)	F	83	49	C3	EF	Fair	32	30.1	39.9	II	Yes
(81)	M	77	97	C2	EF	Poor	43	22.4	30.0	II	Yes
(82)	F	77	82	C1	ORIF	Good	53	22.6	39.8		Yes
(83)	F	21	73	C2	ORIF	Good	100	60.1	49.5		
(84)	M	31	74	B3	ORIF	Good	85	46.2	53.2		
(85)	F	79	68	C1	ORIF	Good	68	47.1	44.6		Yes
(86)	M	48	66	B3	EF	Fair	42	21.3	42.5	II	Yes
(87)	M	64	36	C2	EF	Good	78	56.1	56.9		Yes
(88)	M	23	41	B3	ORIF	Good	81	52.0	41.3		
(89)	M	58	61	B3	EF	Fair	38	19.3	44.8	II	Yes
(90)	M	60	65	C3	ORIF	Good	94	56.4	57.8		Yes
(91)	M	34	59	B2	ORIF	Good	100	54.1	43.4		
(92)	M	45	54	B2	ORIF	Fair	80	37.3	32.1	I	Yes
(93)	M	30	63	C1	ORIF	Good	91	52.3	46.2		
(94)	M	65	43	C1	ORIF	Good	96	56.33	56.9		

Distribution of the type of fracture according to Ovadia and Beals criteria in the analyzed patient series. FW-UP: follow-up, TISS.DAMAGE: tissue damage.

**Table 3:** ^*∗*^In these cases the definitive EF was used due to the severity of tissue damage and bad local skin conditions at the time of trauma.

AO classification	Surgical techniques		Ovadia & Beals criteria			AOFAS	PCS	MCS	Gustilo & Anderson open fracture			Tissue damage
	Type	N°	Good	Fair	Poor				I	II	III	
43-B1	MIPO	3	3	-	-	94.0	57.8	54.5	1	-	-	1
	ORIF	2	2	-	-	87.0	49.2	45.0	-	-	-	-
	EF	-	-	-	-	-	-	-	-	-	-	-

43-B2	MIPO	3	2	1	-	66.7	40.0	42.4	-	-	-	-
	ORIF	6	5	1	-	94.0	52.5	43.6	1	-	-	2
	EF	1^*∗*^	-	1	-	68.0	46.7	36.1	-	-	-	1

43-B3	MIPO	1	-	1	-	60.0	39.8	41.2	-	-	-	-
	ORIF	16	9	6	1	76.3	47.2	43.1	2	-	-	5
	EF	2^*∗*^	-	2	-	40.0	20.3	43.7	-	2	-	2

43-C1	MIPO	2	1	1	-	82.5	49.4	46.2	-	-	-	-
	ORIF	16	15	1	-	81.3	44.2	49.2	-	-	-	5
	EF	1	-	-	1	60.0	38.0	28.0	-	1	-	1

43-C2	MIPO	6	6	-	-	87.0	48.2	50.5	-	-	-	-
	ORIF	15	11	3	1	82.3	44.5	42.7	-	-	-	7
	EF	5	2	1	2	63.8	40.0	43.9	1	2	-	4

43-C3	MIPO	2	2	-	-	86.0	56.1	56.3	-	-	-	2
	ORIF	8	3	5	-	74.8	43.4	54.2	2	-	-	7
	EF	5	-	3	2	36.2	25.1	37.1	-	1	2	4

**Table 4:** Complications recorded in our patient cohort after treatment. N°: number of patients; %: percentage of patients referred to the whole cohort.

Complications	N°; (%)
*Whole cohort*	*39; (41.49)*

*Early complications:*	*20; (21.28)*
(i) Superficial infection	12; (12.77)
(ii) Wound dehiscence	5; (5.32)
(iii) DVT	3; (3.19)

*Late complications:*	*19; (20.21)*
(i) Delayed union	7; (7.44)
(ii) Osteomyelitis	4; (4.26)
(iii) Malunions	3; (3.19)
(iv) Loss of reduction	3; (3.19)
(v) Sudeck syndrome	2; (3.19)
